# Work-Related Exposures Mediate Occupational Class Disparities in SARS-CoV-2 Infection in France

**DOI:** 10.3389/ijph.2026.1608670

**Published:** 2026-02-10

**Authors:** Emilie Counil, Narges Ghoroubi, Myriam Khlat

**Affiliations:** 1 Mortality, Health and Epidemiology Research Unit, Institut National D'études Démographiques (INED), Aubervilliers, France; 2 Institut de Recherche Interdisciplinaire sur les Enjeux Sociaux (IRIS), Institut National de la Santé et de la Recherche Médicale (INSERM) / École des Hautes Études en Sciences Sociales (EHESS), Aubervilliers, France

**Keywords:** COVID-19, EpiCoV, mediation analysis, social inequalities, work-related exposures

## Abstract

**Objectives:**

This study examines SARS-CoV-2 infection by occupational class (OC) among working adults during the early pandemic in France and the mediating role of work-related exposures in regions highly and less affected by COVID-19.

**Methods:**

We analyzed data from 46,849 workers in the French EpiCoV cohort. SARS-CoV-2 infection was defined by self-reported COVID-19-like symptoms between mid-March and the end of June 2020. We related OC with reporting COVID-19-like symptoms in both regions and assessed the mediating effect of work-related exposures using the Karlson-Holm-Breen method of mediation analysis.

**Results:**

During the study period, 7.1% of workers reported COVID-19-like symptoms. In less-affected regions, the highest OC workers reported symptoms more often than the lowest, while in the highly affected regions, middle OCs reported symptoms more often than those in the upper class. Regardless, work-related factors increased symptom risk in the middle and lower OCs compared to the highest OC.

**Conclusion:**

Distinct transmission dynamics shaped the evolution of occupational class disparities during the early pandemic. Workplace exposures played a significant role in these disparities, even when offset by other exposure-related factors.

## Introduction

Research on socio-occupational disparities in SARS-CoV-2 infection during the early pandemic has yielded mixed findings. Some studies have demonstrated higher risk among lower occupational classes (technical occupations, personal service occupations, and plant and machine operatives) compared with managers and senior officers [1], while others have found no significant associations [2] or even an elevated infection risk among workers holding highly complex activities compared with skilled workers [3]. Furthermore, social differences in COVID-19 risk have likely evolved throughout the pandemic. A review found that in most studies conducted in high-income countries up to October 2021, affluent populations initially had higher COVID-19 incidence, which later shifted to disadvantaged groups. Some studies reported stable socioeconomic inequalities, while others observed widening or, rarely, narrowing gaps [4].

This evidence echoes two hypotheses regarding the mechanisms underlying socio-occupational disparities in SARS-CoV-2 infection during the early pandemic. One suggests that COVID-19 inequalities mirrored the well-established social gradient in health [5], consistent with the framework of occupation as a social determinant of health [6]. The other proposes a more dynamic pattern, with, at the start of the pandemic, a higher COVID-19 incidence in upper occupational classes, possibly due to international travel, greater mobility, and more frequent social interactions through leisure activities, before a shift occurred under diverse mitigation contexts [7].

These hypotheses can be tested empirically by focusing on the workforce and contrasting different epidemic backgrounds. Indeed, most studies on the social gradient of COVID-19 have focused on the general population. Although older adults faced the highest risk for COVID-19 complications [8] and mortality [9], working-age adults were among the most infected [10], making it crucial to examine the socio-occupational disparities in infection risk within this group. Besides, focusing on the workforce further allows the disentangling of different exposures driving the epidemic spread in the broader population, as occupational variation in COVID-19 risk [11] could be partially explained by work-related exposures [12]. Workplaces with public or patient interpersonal contact have been shown to increase the odds of SARS-CoV-2 infection [13]. Furthermore, public transportation is considered high-risk, particularly in crowded urban areas where distancing is difficult [14]. Commuting by public transportation to work can, therefore, be regarded as work-related exposure. There was also considerable heterogeneity in the implementation of occupational health and safety measures, although the lack of personal protective equipment, particularly masks, was widespread during the early months of the pandemic [15]. Finally, the time-varying reproduction number of COVID-19, influenced by the background infection rate, spatial virus distribution, and public health interventions [16], likely shaped the importance of different exposure circumstances over time.

In response to the first wave of COVID-19, the French government imposed a strict lockdown starting on March 17, 2020, rated by the Oxford COVID-19 Government Response Tracker [17] as one of the most stringent “lockdown-style” policies. Measures included restrictions on leisure activities, widespread remote work for teleworkable occupations, as well as paid layoffs for non-essential workers and those with certain health conditions [18]. These measures reduced exposure risk for non-essential workers compared to essential in-person workers, who were more often clerical and manual workers than managers [19]. A French cohort study of the general population found higher SARS-CoV-2 antibody prevalence in May 2020 in healthcare workers compared to non-essential workers but not in other essential workers [20]. Based on the same data, another analysis showed that working onsite during the lockdown increased the risk of reporting COVID-19-like symptoms during and right after the first pandemic peak [21]. A French cross-sectional study during the early pandemic underscored how lockdown policies exacerbated pre-existing social inequalities. Privileged groups experienced greater benefits, with a sharper decline in possible COVID-19 cases compared to working-class individuals, especially those engaged in essential, in-person work [22]. Yet, little is known about socio-occupational differences in COVID-19 incidence among the French workforce during the early stages of the pandemic, let alone about the role of work-related exposure factors. The stringent restriction of non-essential social interaction during the first wave may have amplified the contribution of occupational exposures compared to contexts with less stringent lockdowns. However, some activities were still allowed, such as grocery shopping and caregiving [18], and some social interactions likely continued despite legal restrictions. Additionally, the early pandemic was marked by wide spatial disparities in incidence driven by clusters in specific areas, urbanization, and mobility [23]. These spatial disparities in the pre-lockdown epidemic background likely influenced the social patterns of personal vs. work-related contacts and infections during the lockdown, which remains understudied.

The objective of our study is to contribute to filling these knowledge gaps by: (I) examining the occupational class disparities in COVID-19 cumulative incidence among the working population during the first wave of the pandemic in France; (II) assessing the mediating effect of work-related risk factors on this gradient through mediation analysis; and (III) comparing these patterns across regions of France that were highly affected and less affected by the pandemic in March 2020.

## Methods

### Data

The EpiCoV longitudinal cohort sampled individuals aged 15 and older from the national tax register, covering 96% of France’s population. Data on socioeconomic status, migration history, health, work, and living conditions were collected over four waves through self-computer-assisted-web or computer-assisted-telephone interviews. Further details of the study design are described elsewhere [24]. For our analyses, we restricted the sample to workers aged 18 to 64 living in metropolitan France (i.e., excluding the French Overseas Departments) who participated in the first (2 May to 2 June 2020) and second (26 October to 14 December 2020) waves of the cohort (46,849 participants). Supplementary Figure S1 details the selection process.

EpiCoV survey was approved by an ethics committee (Comité de Protection des Personnes Sud Méditerranée III 2020-A01191-38) and France’s National Data Protection Agency (Commission Nationale Informatique et Liberte’s, CNIL, MLD/MFI/AR205138) in April 2020.

### Study Variables

The outcome variable was a binary measure indicating whether participants reported at least one of the COVID-19-like symptoms considered most suggestive of infection by the French Public Health Agency in 2020: any unusual episode of sudden loss of taste or smell, fever with cough, fever with shortness of breath, or fever with chest oppression [25]. The symptom reporting period spanned from the start of the first lockdown in France (17 March 2020) to the end of June, as the lifting of lockdown measures was progressive up to mid-June. Symptoms reported within this time frame, either at the time of the survey or retrospectively, in the first and second study waves were combined to construct this outcome variable.

The exposure variable was occupational class. Participants who reported being employed before the first lockdown, whether part-time or full-time, were classified into four occupational classes following the 2020 French classification of occupations and socio-occupational categories: lower, lower-middle, upper-middle, and upper classes. This variable incorporates both salaried and self-employed workers, differentiating occupations by their qualifications, job types, and contract conditions, providing an updated measure of socio-occupational stratification among the working population [26].

Three work-related exposures to SARS-CoV-2 were included as mediators. Contact with the public and using public transportation to commute to work during the first lockdown were assessed retrospectively in the second EpiCoV wave with questions: “At work during the first lockdown, did you interact face-to-face with the public (e.g., users, patients, travelers, customers)?; “Did you use public transportation (bus, streetcar, metro, train) to get to work or your place of study during the first lockdown?”. Self-perceived work-related exposure was assessed in the first wave by the question “Have you ever feared for your health due to your working conditions related to the coronavirus epidemic since the lockdown began?”.

Confounding variables included sex, migration background, their interaction, and age. The migration background variable divided participants based on their own and their parents’ place of birth and nationality at birth [27].

All analyses were also adjusted for two potential non-occupational exposures, living in densely populated neighborhoods and overcrowded housing, in order to hold constant these extra-professional exposure pathways and thereby isolate the specific mediating role of work-related exposures in the relationship between occupational class and COVID-19-like symptoms. The variable “living in densely populated neighborhoods” separated densely populated municipalities from municipalities of intermediate, low, and very low density, as defined by Eurostat [28], and overcrowded housing was defined as having a total number of bedrooms and living rooms lower than the number of household members.

To capture the potential effect of the regional context of transmission, we stratified our analyses, dividing metropolitan France’s thirteen regions into two groups based on standardized incidence ratios of COVID-19–related hospitalizations during the first COVID-19 wave [23]: highly affected (Île-de-France and Grand-Est) and less affected (Auvergne-Rhône-Alpes, Bourgogne-Franche-Comté, Brittany, Centre-Val de Loire, Corsica, Hauts-de-France, Normandie, Nouvelle-Aquitaine, Occitanie, Pays de la Loire, Provence Alpes Côte d’Azur). Participants were allocated to regions based on their permanent address at inclusion.

The assumed relationships among the study variables are summarized in a directed acyclic graph (Supplementary Figure S2).

### Analytical Strategy

In each regional group, we first assessed the association between occupational class and self-reported COVID-19-like symptoms during the study period. We then employed the Karlson-Holm-Breen (KHB) mediation analysis to delineate the extent to which work-related exposures to SARS-CoV-2 explain socio-occupational differences in self-reported symptoms. This method decomposes the total effect of the exposure on the outcome into a direct effect, i.e., the effect when accounting for the mediating variables, and indirect effects, i.e., the effects through the mediating variables [29]. Using the KHB command [30] in STATA version 14.2, we decomposed the total effect of occupational class on reporting COVID-19-like symptoms into a direct component, i.e., the part of the effect not mediated by the three work-related exposures, conditional on all adjusted variables, and a joint indirect component, i.e., the part of the effect operating through the three work-related exposures.

To further understand the joint indirect effect through which occupational class affects COVID-19-like symptom reporting, we measured the association between occupational class and each work-related exposure factor as well as the association between each factor and COVID-19-like symptom reporting, separately within each regional group. These analyses were performed with R version 4.2.3 using multiple logistic regression.

All analyses were based on complete cases and applied the EpiCoV survey weights [24].

### Sensitivity Analyses

We conducted four sensitivity analyses to check the robustness of our findings. First, we excluded self-perceived work-related exposure to avoid potential bias from individuals’ risk assessments influenced by COVID-19-like symptoms. Second, we restricted the outcome variable to reporting a sudden loss of taste or smell during a similar period, a symptom highly characteristic of COVID-19. Third, we limited symptom reporting to the strict lockdown period (March 17 to May 11, 2020) to assess whether our results were sensitive to job exposures that may have changed during the step-by-step relaxation of lockdown measures. Fourth, we repeated the analysis after excluding healthcare workers to ensure that the observed socio-occupational differences were not driven by their uneven distribution across occupational classes, given the lack of a specific question about face-to-face contact with COVID-19 patients.

## Results

### Sample Description

Workers in less and highly affected regions differed regarding occupational class and migration background, while age and sex distributions were similar. During the first lockdown and the following month (March-June 2020), 7.1% of workers reported COVID-19-like symptoms, rising to 9.8% in highly affected regions and 6.0% in less affected regions. Across France, 41.6% of workers reported face-to-face interactions with the public, 6.6% used public transportation for commuting, and 23.0% reported self-perceived work-related exposure. Face-to-face contact with the public and self-perceived exposure were more common in less affected regions, while public transportation use was higher in highly affected regions (Table 1).

**TABLE 1:** Participants’ distribution across study variables, France, 2020.

Variable			France		Less affected regions		Highly affected regions	
			N	% [CI]	N	% [CI]	N	% [CI]
Outcome	Reported COVD-19-like symptoms	No	43,525	92.9 [92.7–93.1]	31,309	94.0 [93.7–94.3]	12,226	90.2 [89.7–90.7]
		Yes	3,324	7.1 [6.9–7.3]	1984	6.0 [5.7–6.2]	1,330	9.8 [9.3–10.3]
Exposure	Occupational class	Upper	13,367	28.5 [28.1–28.9]	8,150	24.5 [24.0–24.9]	5,183	38.2 [37.4–39.1]
		Upper-middle	12,902	27.5 [27.1–27.9]	9,421	28.3 [27.8–28.8]	3,487	25.7 [25.0–26.5]
		Lower-middle	12,422	26.5 [26.1–26.9]	9,497	28.5 [28.0–29.0]	2,942	21.7 [21.0–22.4]
		Lower	8,159	17.4 [17.1–17.8]	6,226	18.7 [18.3–19.1]	1944	14.3 [13.7–14.9]
Mediators	Face-to-face contact with the public	No	27,342	58.4 [57.9–58.8]	18,719	56.2 [55.7–56.7]	8,605	63.5 [62.7–64.3]
		Yes	19,507	41.6 [41.2–42.1]	14,574	43.8 [43.2–44.3]	4,951	36.5 [35.7–37.3]
	Using public transport to commute to work	No	43,745	93.4 [93.1–93.6]	31,960	96.0 [95.8–96.2]	11,807	87.1 [96.5–87.7]
		Yes	3,104	6.6 [6.4–6.8]	1,333	4.0 [3.8–4.2]	1749	12.9 [12.3–13.5]
	Self-perceived work-related exposure	No	36,052	77.0 [76.6–77.3]	25,224	75.8 [75.3–76.2]	10,818	79.8 [79.1–80.5]
		Yes	10,797	23.0 [22.7–23.4]	8,069	24.2 [23.8–24.7]	2,738	20.2 [19.5–20.9]
Adjustment variable	Living in a densely populated neighborhood	No	28,729	61.3 [60.9–61.8]	24,169	72.6 [72.1–73.1]	4,657	34.4 [33.6–35.2]
		Yes	18,120	38.7 [38.2–39.1]	9,124	27.4 [26.9–27.9]	8,899	65.6 [64.8–66.4]
	Living in an overcrowded housing	No	42,528	90.8 [90.5–91.0]	30,914	92.9 [92.6–31.1]	11,631	85.8 [85.2–86.4]
		Yes	4,321	9.2 [9.0–9.5]	2,379	7.1 [6.9–7.4]	1925	14.2 [13.6–14.8]
	Age	18–24	2,956	6.3 [6.1–6.5]	2,111	6.3 [6.0–6.6]	845	6.2 [5.8–6.6]
		25–34	11,136	23.8 [23.4–24.2]	7,596	22.8 [22.4–23.3]	3,532	26.1 [25.3–26.8]
		35–44	12,494	26.7 [26.3–27.1]	8,940	26.9 [26.4–27.3]	3,555	26.2 [25.5–27.0]
		45–54	13,050	27.9 [27.4–28.3]	9,504	28.5 [28.1–29.0]	3,551	26.2 [25.5–26.9]
		55–64	7,214	15.4 [15.1–15.7]	5,141	15.4 [15.1–15.8]	2073	15.3 [14.7–15.9]
	Sex	Man	23,333	49.8 [49.3–50.3]	16,560	49.7 [49.2–50.3]	6,772	50.0 [49.1–50.8]
		Woman	23,516	50.2 [49.7–50.7]	16,733	50.3 [49.7–50.8]	6,784	50.0 [49.2–50.9]
	Migration background	Mainstream population	37,502	80.0 [79.7–80.4]	28,407	85.3 [84.9–85.7]	9,141	67.4 [66.6–68.2]
		French overseas departments natives	847	1.8 [1.7–1.9]	428	1.3 [1.2–1.4]	414	3.1 [2.8–3.4]
		European immigrants and descendants of immigrant	3,637	7.8 [7.5–8.0]	2,202	6.6 [6.3–6.9]	1,425	10.5 [10.0–11.0]
		Non-european immigrants and descendants of immigrant	4,863	10.4 [10.1–10.7]	2,256	6.8 [6.5–7.1]	2,576	19.0 [18.3–19.7]
Total			46,849	100	33,293	100	13,556	100

All % estimates are weighted.

CI: 95% confidence intervals.

### Main Analysis

During spring 2020, compared with the upper occupational class, the lower class had significantly lower odds of reporting COVID-19-like symptoms in less affected regions, whereas in highly affected regions, the two middle classes had significantly higher odds of reporting such symptoms (Table 2).

**TABLE 2:** Total, direct, and joint indirect effect of occupational class on reporting COVID-19-like symptoms. Mediation analysis for all work-related risk factors, France, 2020.

Occupational class	% [CI] reported COVID-19-like symptoms	Total effect OR^a^ [CI]	Direct effect OR^a^ [CI]	Joint indirect effect OR^a^ [CI]	% Of joint indirect effect^b^		
					Public	Transport	Self-perceived
Less affected regions							
Upper	6.6 [6.1–7.2]	Ref	Ref	Ref	Ref	Ref	Ref
Upper-middle	6.1 [5.7–6.6]	0.93 [0.81–1.07]	0.87 [0.76–1.01]	1.06 [1.04–1.08]^c^	15	−1	86^c^
Lower-middle	5.7 [5.2–6.2]	0.87 [0.76–1.01]	0.82 [0.71–0.95]^c^	1.07 [1.04–1.09]^c^	16	−2	86^c^
Lower	5.3 [4.7–5.8]	0.80 [0.67–0.95]^c^	0.73 [0.61–0.87]^c^	1.09 [1.06–1.11]^c^	14	−1	87^c^
Highly affected regions							
Upper	8.5 [7.8–9.3]	Ref	Ref	Ref	Ref	Ref	Ref
Upper-middle	10.2 [9.2–11.2]	1.21 [1.03–1.43]^c^	1.15 [0.97–1.36]	1.06 [1.02–1.09]^c^	28^c^	−6	78^c^
Lower-middle	11.6 [10.4–12.8]	1.44 [1.19–1.78]^c^	1.34 [1.11–1.63]^c^	1.07 [1.03–1.11]^c^	32^c^	−4	72^c^
Lower	9.9 [8.6–11.3]	1.21 [0.94–1.55]	1.10 [0.85–1.43]	1.09 [1.04–1.14]^c^	26^c^	−8	82^c^

^a^ Adjusted for sex, migration background, their interaction, age, living in densely populated neighborhoods, and living in overcrowded housing.

^b^ Percentage of the joint indirect effect that is mediated by each mediator.

^c^ Results significant at the 5% level (p < 0.05).

OR: odds ratio; CI: 95% confidence intervals; Ref: reference; Public: Face-to-face contact with the public; Transport: Using public transport to commute to work; Self-perceived: Self-perceived work-related exposure.

All estimates are weighted.

Total effect: The overall impact of occupational class on reporting COVID-19-like symptoms; Direct effect: The specific impact of occupational class on reporting COVID-19-like symptoms in the absence of the work-related risk factors; Joint indirect effect: The impact of occupational class on reporting COVID-19-like symptoms that are mediated by work-related risk factors.

Sample sizes: 13,675 participants in less affected regions; 13,556 participants in highly affected regions.

In less affected regions, the total effect of occupational class on reporting COVID-19-like symptoms combined a negative direct effect and a positive joint indirect effect mediated by work-related exposures (Table 2). This suggests that while the lower class reported fewer symptoms overall, work-related factors did increase the odds of reporting symptoms in the lower class relative to the upper one. Factors not included in the mediation analysis must therefore have counterbalanced this effect by increasing the risk of symptom reporting in the upper class compared with the other classes. Analysis of the contribution of each work-related risk factor showed that the mediation was driven solely by self-perceived work-related exposure.

In contrast, in highly affected regions, total, direct, and joint indirect effects for symptom-reporting in the middle classes were positive compared to the upper class (Table 2). This indicates that excess symptoms in the middle classes were partly due to occupational exposure. The joint indirect effect of work-related exposures was also significant in the lower class, suggesting an increase in symptoms due to work-related factors. Self-perceived work-related exposure and, to a lesser extent, face-to-face public contact contributed to this mediation.

To better illustrate how the total effect corresponds to the sum of the direct and joint indirect effects, Supplementary Figure S2 summarizes the KHB mediation results by presenting the underlying coefficients that correspond to the odds ratios reported in Table 2.

These joint indirect effects were further broken down into associations between occupational class and work-related exposures (Table 3) and between each exposure factor and COVID-19-like symptom reporting (Table 4). Both regions showed a social gradient in face-to-face public contact and self-perceived work-related exposure to SARS-CoV-2, with the upper class having the lowest prevalence. In less affected regions, the lower-middle class used public transport the least and the upper class the most, but the prevalence was overall low. Conversely, in highly affected regions, the upper class was less likely to use public transport than other classes (Table 3). Face-to-face public contact and self-perceived work-related exposure were linked to higher symptom reporting in both regions. Commuting by public transport increased symptom-reporting odds only in less affected regions (Table 4).

**TABLE 3:** The association between occupational class and work-related exposure to SARS-CoV-2, France, 2020.

Occupational class	Face-to-face contact with the public		Using public transport to commute to work		Self-perceived work-related exposure	
	% Exposed [CI]	OR^a^ [CI]	% Exposed [CI]	OR^a^ [CI]	% Exposed [CI]	OR^a^ [CI]
Less affected regions						
Upper	34.0 [33.0–35.1]	Ref	4.9 [4.4–5.4]	Ref	14.4 [13.6–15.2]	Ref
Upper-middle	45.2 [44.2–46.2]	1.57 [1.47–1.68]^b^	4.0 [3.6–4.4]	0.91 [0.76–1.07]	26.1 [25.2–27.0]	2.05 [1.89–2.23]^b^
Lower-middle	47.5 [46.5–48.5]	1.71 [1.59–1.83]^b^	3.2 [2.9–3.6]	0.79 [0.66–0.96]^b^	27.0 [26.1–27.9]	2.14 [1.97–2.34]^b^
Lower	48.7 [47.4–49.9]	1.81 [1.66–1.97]^b^	4.0 [3.5–4.5]	0.84 [0.67–1.05]	30.1 [28.9–31.2]	2.58 [2.33–2.83]^b^
Highly affected regions						
Upper	26.1 [24.9–27.3]	Ref	11.3 [10.4–12.2]	Ref	10.5 [9.7–11.4]	Ref
Upper-middle	39.9 [38.3–41.6]	1.85 [1.67–2.05]^b^	13.3 [12.2–14.5]	1.35 [1.15–1.58]^b^	24.0 [22.6–25.5]	2.64 [2.31–3.02]^b^
Lower-middle	45.0 [43.2–46.8]	2.27 [2.02–2.55]^b^	12.2 [11.1–13.5]	1.22 [1.02–1.47]^b^	25.2 [23.7–26.9]	2.88 [2.48–3.34]^b^
Lower	45.5 [43.3–47.8]	2.37 [2.04–2.76]^b^	17.5 [15.8–19.3]	1.66 [1.32–2.09]^b^	31.6 [29.6–33.7]	4.10 [3.42–4.91]^b^

^a^ Adjusted for sex, migration background, their interaction, age, living in densely populated neighborhoods, and living in overcrowded housing.

^b^ Results significant at the 5% level (p < 0.05).

OR: odds ratio; CI: 95% confidence intervals; Ref: reference.

All estimates are weighted.

Sample sizes: 13,675 participants in less affected regions; 13,556 participants in highly affected regions.

**TABLE 4:** The association between work-related exposure to SARS-CoV-2 and reporting COVID-19-like symptoms, France, 2020.

Exposure factors		% [CI] reported COVID-19-like symptoms	OR^a^ [CI]
Less affected regions			
Face-to-face contact with the public	No	5.5 [5.2–5.8]	Ref
	Yes	6.6 [6.2–7.0]	1.23 [1.10–1.37]^b^
Using public transport to commute to work	No	5.9 [5.6–6.1]	Ref
	Yes	8.0 [6.6–9.6]	1.27 [1.00–1.63]^b^
Self-perceived work-related exposure	No	5.3 [5.0–5.5]	Ref
	Yes	8.1 [7.6–8.8]	1.59 [1.42–1.79]^b^
Highly affected regions			
Face-to-face contact with the public	No	9.1 [8.5–9.8]	Ref
	Yes	11.0 [10.1–11.9]	1.24 [1.08–1.43]^b^
Using public transport to commute to work	No	9.8 [9.3–10.4]	Ref
	Yes	9.8 [8.5–11.3]	0.96 [0.78–1.19]
Self-perceived work-related exposure	No	9.1 [8.5–9.6]	Ref
	Yes	12.8 [11.6–14.1]	1.49 [1.27–1.74]^b^

^a^ Adjusted for sex, migration background, their interaction, age, living in densely populated neighborhoods, and living in overcrowded housing.

^b^ Results significant at the 5% level (p < 0.05).

OR: odds ratio; CI: 95% confidence intervals; Ref: reference.

All estimates are weighted.

Sample sizes: 13,675 participants in less affected regions; 13,556 participants in highly affected regions.

### Sensitivity Analyses

In all four sensitivity analyses, the joint indirect effects of work-related exposure remained significant and positive in both less and highly affected regions, indicating that work-related exposure consistently placed middle and lower occupational classes at greater risk of reporting COVID-19-like symptoms compared with the upper occupational class. Regarding the gradient in reporting COVID-19-like symptoms by occupational class, whether restricting the definition of symptoms to sudden loss of taste or smell, or limiting the observation period to the strict lockdown, no differences in reporting were observed across occupational classes in less affected regions. By contrast, in highly affected regions, the middle and lower occupational classes reported more symptoms than the upper class. Finally, after excluding healthcare workers, the lower-middle and lower occupational classes reported fewer symptoms than the upper class in less affected regions, whereas the pattern remained consistent with the main analysis in highly affected regions (Supplementary Tables S1–S4).

## Discussion

### Key Findings

Our study identified different patterns of occupational class disparities among working adults depending on the local spread of the pandemic in France during the spring of 2020. In regions that were less affected pre-lockdown, COVID-19-like symptom reporting was higher among upper-class workers, while in regions that were already strongly hit, middle-class workers reported symptoms more frequently than upper-class, even after adjusting for known confounders. Another key finding was that despite these regional differences, work-related exposures systematically increased the likelihood of symptom reporting among middle and lower-class workers compared to those in the higher class, hence positively mediating the association between occupational class and risk of infection. However, commuting to work by public transportation did not contribute to this mediation.

### Interpretation

Few studies have explored the mediating role of work-related exposures in occupational class differences in COVID-19 risk, even less in the early pandemic. Yet, the stringent lockdowns implemented during the first pandemic wave involved large social disparities in the ability to work remotely or otherwise stay on compensated layoff among the French workforce [31]. A study on SARS-CoV-2 infections in the German working population reported a significant contribution of remote work to the relationship between higher education and lower risk of infection from March 2020 to January 2021 [32]. This mediating role was shown in a classical social gradient similar to that in regions already highly affected pre-lockdown in France. A UK study later found higher odds of infection-related seropositivity from February to June 2021 among healthcare workers, indoor trade, process, and plant workers, leisure and personal service workers, and transport and mobile machine operatives, compared with workers in other professional and associate occupations. Work-related close contacts mediated a substantial part of these associations, even though some workers in high-risk occupations had residual risks, suggesting that other factors also contributed [33].

These findings were based on a cumulative infection risk through the first and second waves of the pandemic in England and Wales, from its onset to June 2021, and, therefore, cannot be directly compared with our study conducted over the first epidemic wave in France. Moreover, they focus on specific occupational groups with no direct social hierarchy, contrary to our grouping by ordered occupational class. Nevertheless, they are consistent with and complementary to ours, suggesting that work-related exposures remained important mediators between occupation and infection risk throughout the pandemic.

Among the three work-related exposures examined, workers in all occupational classes reported higher levels of face-to-face public contact and self-perceived work-related exposure compared with the upper class, and both factors were associated with greater odds of reporting COVID-19-like symptoms. This reflects a socially patterned distribution of work-related risk factors. In contrast, commuting by public transport did not contribute to the social gradient in COVID-19 across occupational classes. In less affected regions, this likely reflects the very small proportion of workers who used public transport during lockdown. In highly affected regions, commuting by public transport was not associated with symptom reporting. This result should be interpreted in light of reinforced disinfection protocols and the sharp drop in the number of people using public transport, which may have limited transmission risk in this setting. Further research will be necessary to assess this risk in later stages of the pandemic, particularly during periods outside lockdown.

Research investigating occupational class differences in COVID-19 risk at a single point in time during the early pandemic has reported mixed findings [1–3], likely due to differences in epidemic background, geographic contexts, and pandemic control strategies. To our knowledge, no previous work has examined differences in infection by occupational class among workers in France according to the pre-lockdown outbreak stage, reflected in our study by the distinction we made between highly and less affected regions. These pre-lockdown differences likely involved different transmission patterns over time and space, reflecting the relative weight of social contacts through personal/leisure and business/work activities. These transmission patterns explain the distinctive social pattern of infection that we found in highly compared to less affected regions.

Previous work confirmed the hypothesis that SARS-CoV-2 incidence was initially higher among upper classes in France but decreased progressively over time [7, 22]. A study from Hong Kong illustrated a shift in socioeconomic disparities from January to August 2020. Executives and professionals initially had higher infection rates, likely due to increased mobility and social interactions abroad. However, the trend reversed by the summer, with production workers and foreign domestic helpers experiencing higher rates. This shift was attributed to executives working from home, which reduced their exposure [34].

A French study based on the same cohort as ours, but including both workers and inactive, examined the association between socio-occupational categories and self-reported anosmia or ageusia from May to June 2020. The authors found that low-skilled employees and manual workers reported significantly less anosmia or ageusia during the first epidemic peak compared to senior executives. However, after the peak, only middle executive professionals were at increased risk, and less privileged occupational categories showed no significant differences with senior executives [21]. This pattern likely reflects the virus’s initial spread originating from the upper classes, primarily through business and leisure activities, before disseminating to the broader population, with a faster spread in densely populated areas with overcrowded housing. The lockdown protected both upper-class workers who could telework and some lower-skilled workers on paid layoff. In contrast, frontline and essential workers faced greater risks due to continued workplace exposure and contact with colleagues, the public, and infected patients [31].

This pattern aligns with what we observed in regions already hard hit when the first lockdown was implemented nationally in France but differs from what we observed in the less affected regions, where upper-class workers remained at higher risk of infection despite the significant contribution of work-related exposures among all workers. One explanation could be the specific epidemic background in less affected regions, many of which were more modestly hit throughout the first wave. Although all regions experienced a rapid rise, peak, and decline in COVID-19-related hospitalizations and deaths [23], this comparatively lower background rate may have influenced risk perception and behaviour, particularly among upper-class workers who were more likely to maintain social contacts and less likely to comply with other distancing and protective measures, as observed in the UK [35]. On the other hand, assessing the role of workplace exposures among middle and lower-class workers was more difficult in this epidemic context, as infection risk through work depended on both contact intensity in high-risk workplaces and background infection rates. Our mediation analysis provided original evidence on the contribution of work-related exposures, even in low-intensity backgrounds where middle and/or lower classes were less affected overall.

### Strengths and Limitations

A large sample, representative of the French population, enabling detailed analysis of the working population during the early pandemic was a major strength of our study. In addition, using self-reported symptoms led to precise identification of infection timing, often missing in serological tests. We examined SARS-CoV-2 infection as the outcome, not COVID-19 mortality, as lethality greatly depends on demographic and pre-existing health conditions [36]. Additionally, the study provides self-reported data on individual working conditions, going beyond estimates from job titles, such as job exposure matrices based on expert assessment. Lastly, consistent results across sensitivity analyses confirm the robustness of our findings.

This study, however, has certain limitations. First, identifying infection based on self-reported symptoms is challenging. Socially advantaged people have been shown to report health issues more often [37] but are more likely to be asymptomatic, as severe symptoms have been reported less frequently in high-income neighborhoods [38]. Thus, the direction of bias remains unclear. Secondly, the role of occupational exposure in infection risk may have been underestimated. Ideally, only those who developed symptoms from 1 week after the start to 1 week after the end of the lockdown would have been included, given COVID-19’s six-day incubation period on average [39]. However, the questionnaire lacked this specificity. For instance, teachers exposed pre-lockdown but working remotely during it might report no exposure, even if they were infected at work. Additionally, the simultaneous collection of exposure and infection data may have led symptomatic workers on sick leave to report symptoms but not workplace exposure, further underestimating the role of occupational exposure. Another limitation is the potential correlation between mediators, which may challenge the assumption of independence required to estimate the joint indirect effect. For example, taking public transportation to work could influence self-perceived work-related exposure. Moreover, as participants were classified into highly or less affected regions based on their place of residence, some degree of misclassification is possible for individuals who commuted across regional borders for work. These individuals represent a small proportion of the regions’ populations, and furthermore, this type of misclassification is likely to dilute regional differences in infection risk. Finally, our overall approach to work-related infection ignores that part of the so-called family-acquired or community-acquired infections may happen through an index case contaminated through work [40].

### Conclusion

This study supports existing evidence that work-related risk factors drive disparities, increasing COVID-19 risk among lower and intermediate occupational classes, independent of overall social patterns. Understanding occupational transmission is crucial for grasping community spread dynamics and guiding targeted control strategies, such as prioritizing vaccination based on occupational risk.
