# Supplementary material for: Work-Related Exposures Mediate Occupational Class Disparities in SARS-CoV-2 Infection in France
Source: Int J Public Health. 2026 Feb 10;71:1608670. doi: 10.3389/ijph.2026.1608670 (PMC12929173; doi:10.3389/ijph.2026.1608670)
Supplement: Supplementary file 1 [file Supplementaryfile1.docx]

# International Journal of Public Health

# Title: Work-related exposures mediate occupational class disparities in SARS-CoV-2 infection in France

# Supplementary material

Supplementary Figure 1. Study sample selection from the EpiCoV cohort


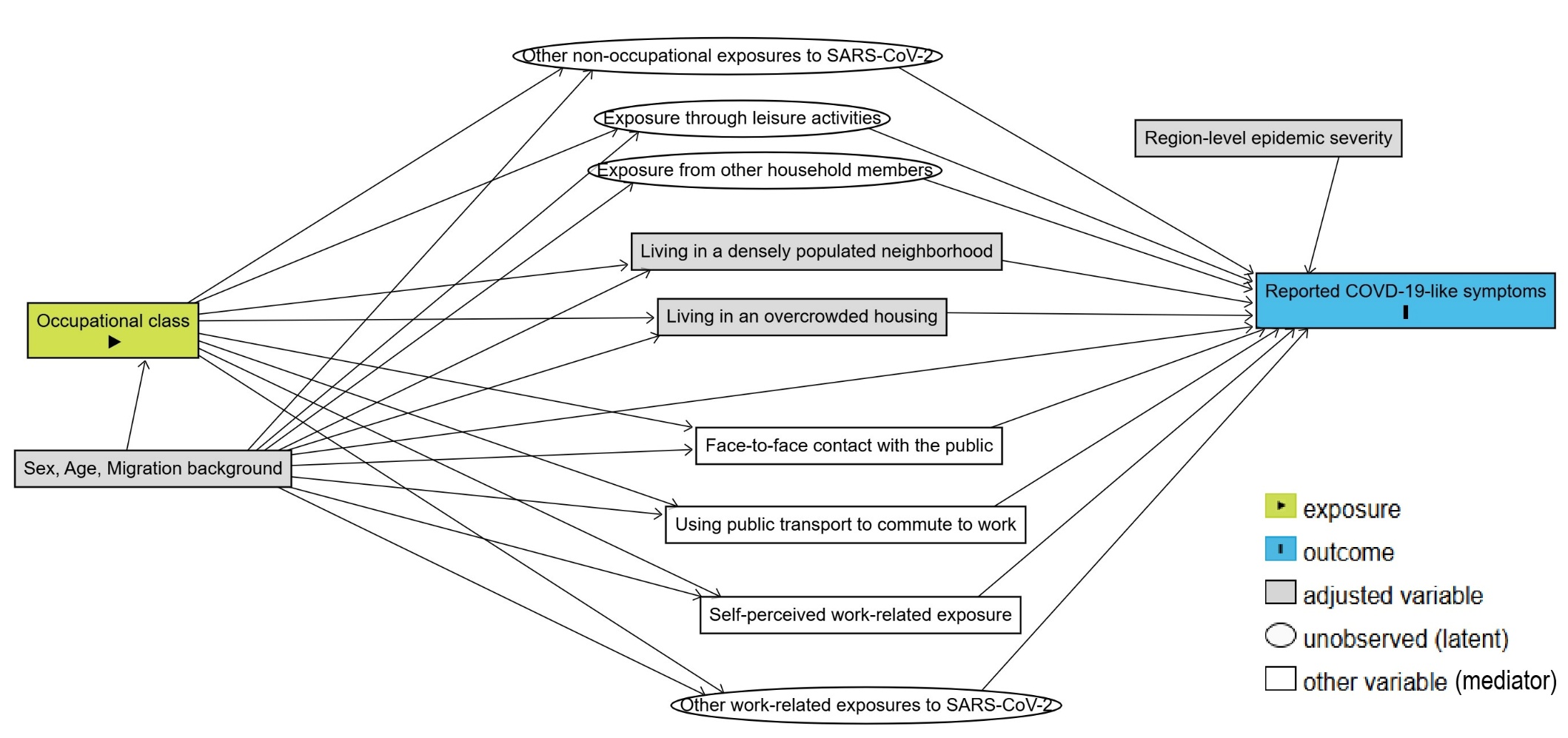


Supplementary Figure 2. Directed acyclic graph (DAG) illustrating the assumed relationships among occupational class (exposure), work-related exposures (mediators), self-reported COVID-19-like symptoms (outcome), and confounders.


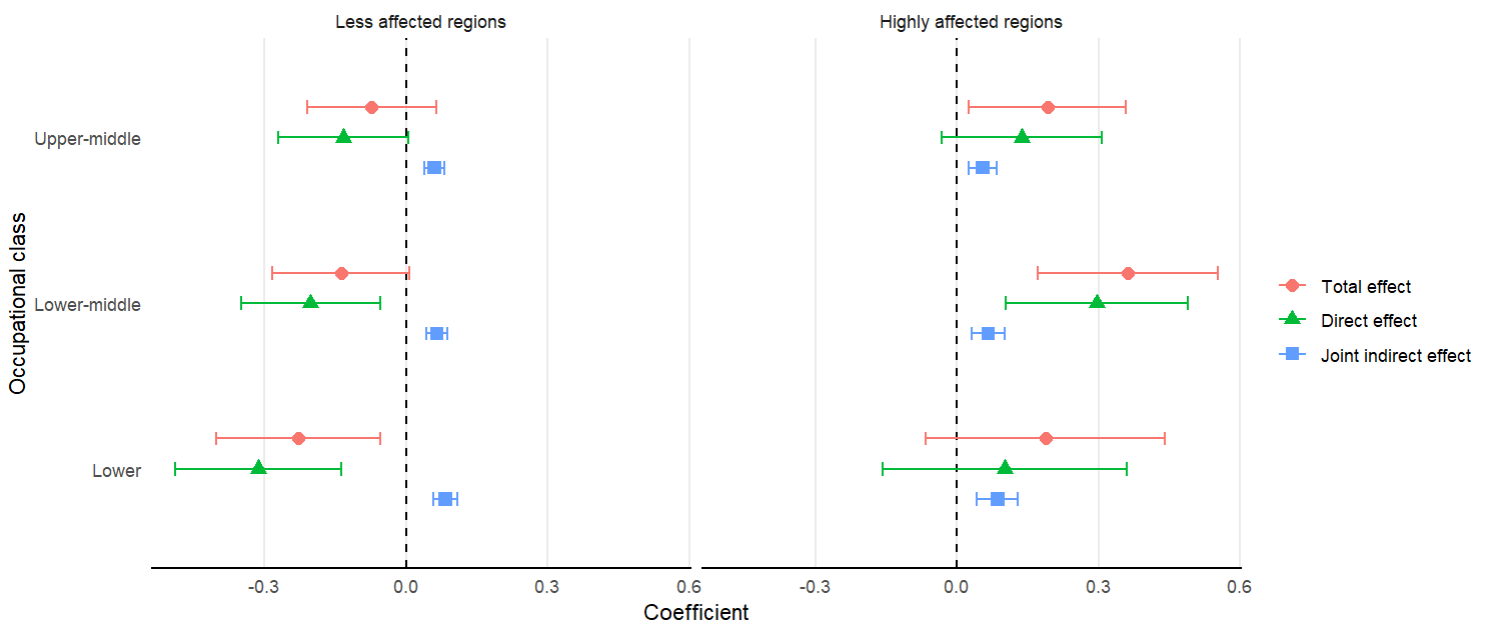


Supplementary Figure 3. Coefficients of the total, direct, and joint indirect effect of occupational class on reporting COVID-19-like symptoms. Mediation analysis for all work-related risk factors, France, 2020

Supplementary table 1. Total, direct, and joint indirect effect of occupational class on reporting COVID-19-like symptoms. Mediation analysis for all work-related risk factors except for the self-perceived exposure, France, 2020

| Occupational class | % [CI] reported COVID-19-like symptoms | Total effect  OR^1^ [CI] | Direct effect  OR^1^ [CI] | Joint indirect effect  OR^1^ [CI] | % of joint indirect effect | |
| --- | --- | --- | --- | --- | --- | --- |
|  |  |  |  |  | Public | Transport |
| Less affected regions | | | | | | |
| Upper | 6.6 [6.1-7.2] | Ref | Ref | Ref | Ref | Ref |
| Upper-middle | 6.1 [5.7-6.6] | 0.94 [0.82-1.07] | 0.91 [0.80-1.05] | 1.02 [1.01-1.04]^*^ | 103^*^ | -3 |
| Lower-middle | 5.7 [5.2-6.2] | 0.88 [0.76-1.01] | 0.85 [0.74-0.99]^*^ | 1.03 [1.01-1.04]^*^ | 106^*^ | -6 |
| Lower | 5.3 [4.7-5.8] | 0.80 [0.67-0.95]^*^ | 0.78 [0.65-0.93]^*^ | 1.03 [1.01-1.05]^*^ | 104^*^ | -4 |
| Highly affected regions | | | | | | |
| Upper | 8.5 [7.8-9.3] | Ref | Ref | Ref | Ref | Ref |
| Upper-middle | 10.2 [9.2-11.2] | 1.22 [1.03-1.43]^*^ | 1.19 [1.01-1.40]^*^ | 1.02 [1.00-1.05]^*^ | 116^*^ | -16 |
| Lower-middle | 11.6 [10.4-12.8] | 1.44 [1.19-1.74]^*^ | 1.39 [1.15-1.69]^*^ | 1.03 [1.01-1.06]^*^ | 107^*^ | -7 |
| Lower | 9.9 [8.6-11.3] | 1.21 [0.94-1.56] | 1.17 [0.91-1.52] | 1.03 [1.00-1.06]^*^ | 121^*^ | -21 |

^*^Results significant at the 5% level (p < 0.05).

1-Adjusted for sex, migration background, their interaction, age, living in densely populated neighborhoods, and living in overcrowded housing.

OR: odds ratio; CI: 95% confidence intervals; Ref: reference; Public: Face-to-face contact with the public; Transport: Using public transport to commute to work.

All estimates are weighted.

Total effect: The overall impact of occupational class on reporting COVID-19-like symptoms; Direct effect: The specific impact of occupational class on reporting COVID-19-like symptoms in the absence of the work-related risk factors; Joint indirect effect: The impact of occupational class on reporting COVID-19-like symptoms that are mediated by work-related risk factors.

Supplementary table 2. Total, direct, and joint indirect effect of occupational class on reporting loss of taste or smell. Mediation analysis for all work-related risk factors, France, 2020

| Occupational class | % [CI] reported loss of taste or smell | Total effect  OR^1^ [CI] | Direct effect  OR^1^ [CI] | Joint indirect effect  OR^1^ [CI] | % of joint indirect effect | | |
| --- | --- | --- | --- | --- | --- | --- | --- |
|  |  |  |  |  | Public | Transport | Self-perceived |
| Less affected regions | | | | | | | |
| Upper | 2.1 [1.8-2.5] | Ref | Ref | Ref | Ref | Ref | Ref |
| Upper-middle | 2.2 [1.9-2.5] | 1.03 [0.82-1.28] | 0.95 [0.76-1.19] | 1.08 [1.05-1.11]^*^ | 23 | -1 | 78^*^ |
| Lower-middle | 1.9 [1.7-2.2] | 0.90 [0.71-1.14] | 0.83 [0.65-1.05] | 1.09 [1.05-1.12]^*^ | 25 | -2 | 76^*^ |
| Lower | 2.0 [1.6-2.3] | 0.89 [0.67-1.19] | 0.80 [0.60-1.06] | 1.11 [1.07-1.15]^*^ | 22 | -1 | 79^*^ |
| Highly affected regions | | | | | | | |
| Upper | 3.9 [3.4-4.5] | Ref | Ref | Ref | Ref | Ref | Ref |
| Upper-middle | 5.4 [4.7-6.2] | 1.40 [1.11-1.76]^*^ | 1.34 [1.06-1.69]^*^ | 1.05 [1.01-1.09]^*^ | 48^*^ | -16 | 68^*^ |
| Lower-middle | 5.5 [4.7-6.4] | 1.43 [1.11-1.82]^*^ | 1.34 [1.05-1.72]^*^ | 1.06 [1.02-1.11]^*^ | 51^*^ | -9 | 58^*^ |
| Lower | 6.3 [5.3-7.5] | 1.55 [1.12-2.13]^*^ | 1.44 [1.04-2.00]^*^ | 1.07 [1.01-1.14]^*^ | 46^*^ | -20 | 74^*^ |

^*^Results significant at the 5% level (p < 0.05).

1-Adjusted for sex, migration background, their interaction, age, living in densely populated neighborhoods, and living in overcrowded housing.

OR: odds ratio; CI: 95% confidence intervals; Ref: reference; Public: Face-to-face contact with the public; Transport: Using public transport to commute to work; Self-perceived: Self-perceived work-related exposure.

All estimates are weighted.

Total effect: The overall impact of occupational class on reporting a loss of taste or smell; Direct effect: The specific impact of occupational class on reporting a loss of taste or smell in the absence of the work-related risk factors; Joint indirect effect: The impact of occupational class on reporting a loss of taste or smell that is mediated by work-related risk factors.

Supplementary table 3. Total, direct, and joint indirect effect of occupational class on reporting COVID-19-like symptoms during the strict lockdown (March 17 to May 11, 2020). Mediation analysis for all work-related risk factors, France, 2020

| Occupational class | % [CI] reported COVID-19-like symptoms | Total effect  OR^1^ [CI] | Direct effect  OR^1^ [CI] | Joint indirect effect  OR^1^ [CI] | % of joint indirect effect | | |
| --- | --- | --- | --- | --- | --- | --- | --- |
|  |  |  |  |  | Public | Transport | Self-perceived |
| Less affected regions | | | | | | | |
| Upper | 5.8 [5.3-6.3] | Ref | Ref | Ref | Ref | Ref | Ref |
| Upper-middle | 5.9 [5.4-6.3] | 1.02 [0.88-1.18] | 0.97 [0.84-1.12] | 1.05 [1.03-1.07]^*^ | 13 | -1 | 88^*^ |
| Lower-middle | 5.4 [5.0-5.9] | 0.96 [0.82-1.11] | 0.91 [0.78-1.06] | 1.05 [1.03-1.08]^*^ | 14^*^ | -2 | 88^*^ |
| Lower | 4.9 [4.4-5.5] | 0.87 [0.72-1.04] | 0.81 [0.67-0.97]^*^ | 1.06 [1.02-1.09]^*^ | 12 | -1 | 89^*^ |
| Highly affected regions | | | | | | | |
| Upper | 8.4 [7.6-9.1] | Ref | Ref | Ref | Ref | Ref | Ref |
| Upper-middle | 9.9 [8.9-10.9] | 1.20 [1.01-1.42]^*^ | 1.13 [0.95-1.34] | 1.05 [1.02-1.08]^*^ | 24^*^ | -6 | 82^*^ |
| Lower-middle | 10.9 [9.8-12.1] | 1.36 [1.12-1.65]* | 1.28 [1.05-2.56]^*^ | 1.07 [1.02- 1.11]^*^ | 27^*^ | -3 | 76^*^ |
| Lower | 9.6 [8.3-11.0] | 1.28 [1.00-1.63]* | 1.08[0.83-1.41] | 1.09 [1.04-1.14]^*^ | 22^*^ | -7 | 85^*^ |

^*^Results significant at the 5% level (p < 0.05).

1-Adjusted for sex, migration background, their interaction, age, living in a densely populated neighborhood, and living in overcrowded housing.

OR: odds ratio; CI: 95% confidence intervals; Ref: reference; Public: Face-to-face contact with the public; Transport: Using public transport to commute to work; Self-perceived: Self-perceived work-related exposure.

All estimates are weighted.

Total effect: The overall impact of occupational class on reporting COVID-19-like symptoms; Direct effect: The specific impact of occupational class on reporting COVID-19-like symptoms in the absence of the work-related risk factors; Joint indirect effect: The impact of occupational class on reporting COVID-19-like symptoms that are mediated by work-related risk factors.

Supplementary table 4. Total, direct, and joint indirect effect of occupational class on reporting COVID-19-like symptoms among non-healthcare workers. Mediation analysis for all work-related risk factors, France, 2020

| Occupational class | % [CI] reported COVID-19-like symptoms | Total effect  OR^1^ [CI] | Direct effect  OR^1^ [CI] | Joint indirect effect  OR^1^ [CI] | % of joint indirect effect | | |
| --- | --- | --- | --- | --- | --- | --- | --- |
|  |  |  |  |  | Public | Transport | Self-perceived |
| Less affected regions | | | | | | | |
| Upper | 6.4 [5.9-7.0] | Ref | Ref | Ref | Ref | Ref | Ref |
| Upper-middle | 6.0 [5.5-6.5] | 0.94 [0.81-1.10] | 0.90 [0.78-1.05] | 1.05 [1.03-1.07]^*^ | 16 | 0 | 84^*^ |
| Lower-middle | 5.3 [4.8-5.8] | 0.84 [0.72-0.98]^*^ | 0.79 [0.68-0.93]^*^ | 1.06 [1.04-1.09]^*^ | 17 | -2 | 85^*^ |
| Lower | 5.1 [4.5-5.7] | 0.79 [0.66-0.95]^*^ | 0.72 [0.60-0.87]^*^ | 1.09 [1.06-1.12]^*^ | 16 | -1 | 85^*^ |
| Highly affected regions | | | | | | | |
| Upper | 8.4 [7.6-9.2] | Ref | Ref | Ref | Ref | Ref | Ref |
| Upper-middle | 9.9 [8.9-11.0] | 1.22 [1.02-1.45]^*^ | 1.17 [0.98-1.40] | 1.04 [1.01-1.07]^*^ | 40^*^ | -18 | 78^*^ |
| Lower-middle | 10.8 [9.7-12.1] | 1.38 [1.13-1.69]^*^ | 1.31 [1.06-1.61]^*^ | 1.06 [1.02-1.10]^*^ | 43^*^ | -7 | 64^*^ |
| Lower | 9.7 [8.4-11.2] | 1.21 [0.93-1.58] | 1.12 [0.86-1.47] | 1.08 [1.02-1.13]^*^ | 38^*^ | -16 | 78^*^ |

^*^Results significant at the 5% level (p < 0.05).

1-Adjusted for sex, migration background, their interaction, age, living in a densely populated neighborhood, and living in overcrowded housing.

OR: odds ratio; CI: 95% confidence intervals; Ref: reference; Public: Face-to-face contact with the public; Transport: Using public transport to commute to work; Self-perceived: Self-perceived work-related exposure.

All estimates are weighted.

Total effect: The overall impact of occupational class on reporting COVID-19-like symptoms; Direct effect: The specific impact of occupational class on reporting COVID-19-like symptoms in the absence of the work-related risk factors; Joint indirect effect: The impact of occupational class on reporting COVID-19-like symptoms that are mediated by work-related risk factors.
